# Supplementary material for: Deletion of wfs1 Impairs Oligodendrocyte Precursor Cells Dorsal Distribution and Myelination Through the wfs1-hmgcs1 Axis in Zebrafish
Source: Biology (Basel). 2026 Aug 16;15(16):1402. doi: 10.3390/biology15161402 (PMC13510151; doi:10.3390/biology15161402)
Supplement: Supplementary file 1 [file biology-15-01402-s001.zip › File S1-Transcriptome sequencing data and differentially expressed genes (DEGs) in wfs1-- zebrafish at 5 dpf/2.KEGG/wfs1KOvsWT/UP/src/dre03008.html]

dre03008


- dre:492761

- Up regulated genes

492761\_1(1.47652845202268)

- dre:393107

- Up regulated genes

393107\_2(6.27903347477089) 393107\_1(6.91276896472144)

- dre:553787

- Up regulated genes

553787\_1(3.49455496027498)

- dre:407084

- Up regulated genes

407084\_1(2.33720346952341)

Close
